# Bridging the Gap: Parents’ Knowledge of Childhood Developmental Milestones and the Early Identification of Children With Developmental Delay

**DOI:** 10.7759/cureus.48232

**Published:** 2023-11-03

**Authors:** Haitham M Alghamdi, Bsaim A Altirkistani, Rabea A Baatya, Yasir O Marghalani, Nahla M Alshaikh

**Affiliations:** 1 College of Medicine, King Saud Bin Abdulaziz University for Health Sciences, Jeddah, SAU; 2 College of Medicine, King Abdullah International Medical Research Center, Jeddah, SAU; 3 Pediatric Neurology, King Abdulaziz Medical City, Jeddah, SAU

**Keywords:** development pediatric, global developmental delay (gdd), developmental and behavioral delay, developmental milestones red flags, presentational delay, parents knowledge, parents’ knowledge, early childhood development, pediatric milestones

## Abstract

Background

Monitoring children's acquisition of developmental milestones is integral to pediatric practice. Though pediatricians are responsible for evaluating children’s development, parents have a crucial role in addressing delays as early as possible, where early detection of developmental delay can help in early intervention and ultimately potentiate a child’s cognitive and social abilities toward an independent life. This study assesses parental knowledge of the warning signs denoting delayed developmental milestone acquisition, in addition to analyzing demographic variables that may influence their level of knowledge.

Methods

This cross-sectional study included 376 parents of children attending pediatric clinics in National Guard Health Affairs- King Abdulaziz Medical City, in Jeddah, Saudi Arabia. A two-section structured questionnaire was utilized. It included 16 option-based questions with one correct answer, while the other options were either an under or overestimate of the age at which the child should acquire a particularly significant milestone development across different domains. A score of 10 out of 16 was chosen as the minimum to show the appropriate level of knowledge.

Results

Most participants (n=282; 75%) were women, and 174 (46.27%) were between 29 and 39 years old. The highest reported level of education was college or higher (n=214; 56.91%). Only 41 (11%) parents had the required level of knowledge, while the remaining 335 (89%) fell short of meeting the passing level (mean 6.59, SD= 2.72). The motor domain had the highest level of accuracy, followed closely behind the cognitive domain. The language and social domains exhibited lower levels of accuracy.

Conclusions

Despite the majority of parents in this group possessing a college education and availing multiple health resources, there is a significant gap in their knowledge of typical trajectories of development milestones. Thus, there is a need for a nationwide initiative to promote the parent's proactive role in monitoring their children's growth.

## Introduction

Child development is a crucial aspect of human growth, encompassing various physical, intellectual, language, emotional, and social domains [1,2]. The first few years of a child's life play a pivotal role in molding their behavior, cognitive abilities, and overall well-being [1-3]. To assess child development accurately, it is imperative to evaluate their developmental milestones, which entail age-specific tasks and functional skills covering five domains: gross motor, fine motor, language, cognitive, and social skills.

Children are generally expected to attain their developmental milestones at certain age limits, and normative data exist; for instance, a child must be able to sit unsupported by six months [4]. Therefore, the uneven acquisition of milestones may alert the physician of a possible developmental disability, warranting further investigation [2,4]. Various diseases that impact the nervous system can cause developmental delays or deviations from the norm. In fact, research indicates that neurological disorders are associated with a delay in walking in 56% of children, with cerebral palsy being the most common suspected or definitive condition [5].

Monitoring children’s acquisition of developmental milestones is an integral part of the pediatric practice where early detection of developmental delay is paramount to enrolling children in early intervention programs, which ultimately improves a child’s cognitive and social abilities toward an independent life [6]. Nevertheless, parents have a crucial role in addressing delays as early as possible by understanding their child’s normal developmental progress and seeking medical attention when necessary. This proactive approach can help pave the way for an independent life.

The caregivers’ understanding of their children’s peculiarities and the provision of a nurturing environment is essential to promote the integral development of their children. Many factors can contribute to a parent's ability to promote their child's growth [7]. Research shows that socioeconomic status, particularly a parent's education level, consistently impacts a child's early developmental skills. Parents who are more educated and economically advantaged tend to have children with stronger vocabulary skills and better cognitive, social, and emotional development [8]. Other studies have explored additional factors, such as where a family lives, access to resources, and past parenting experience, but results vary based on the specific context of each study [7].

From personal experiences and the opinions of local experts in Saudi Arabia, it has been observed that there is a noticeable presentational delay in children with neurological diseases. This poses a crucial challenge to advancing children's health in the country. To address this issue, our study aims to assess parental knowledge of the warning signs denoting delayed developmental milestone acquisition. Furthermore, this study explores potential associations between this knowledge and several variables. The insight gained from this study will help improve our understanding of the current national landscape of most neurological disorders where early diagnosis positively impacts outcomes.

## Materials and methods

This cross-sectional study included parents of children attending pediatric clinics in National Guard Health Affairs- King Abdulaziz Medical City, in Jeddah, Saudi Arabia. Parents who refused to consent to fill out the survey were excluded. The study was approved by the Institutional Review Board at King Abdullah International Medical Research Center (study number: NRJ22J/168/06, approval number IRB/1687/22, dated August 25, 2022).

Study tool

A two-section structured questionnaire was utilized. The first section included demographics such as gender, age of parents, level of education, residency, working status, relationship status, family income per month, presence of a child with special needs, and presence of a helper at home. The second section of this questionnaire included 16 questions that focused on significant developmental milestones over different domains. These domains include gross and fine motor skills (five questions), Language and speech development (five questions), Cognitive development (three questions), and social skills (three questions). Each question has multiple options, with only one being the correct answer. The other options either overestimate or underestimate the age at which a child should achieve that milestone. The questionnaire underwent thorough scrutiny by experts in the field to establish its validity and comprehensibility. Moreover, a preliminary pilot study was executed on a small group of parents before data gathering to validate the questions' lucidity and clarity. Feedback on the questions' comprehensibility was obtained, and the necessary adjustments were made to the questionnaire accordingly. To determine whether parents had an appropriate level of knowledge, each correct answer was awarded a score of 1, resulting in a total score of 16. If a parent answered at least 10 out of 16 questions correctly (equivalent to 62.5%), they were deemed to have an appropriate level of knowledge.

Statistical analysis

Categorical variables were presented as N (%) while numerical variables were presented as either median or mean as appropriate. The chi-square test was used for the comparison of two categorical variables. Statistical significance was defined as P<0.05. Statistical analyses were performed using JMP Statistical Software version 15.2.0 (SAS Institute, Cary, North Carolina, United States).

## Results

A total of 376 parents participated in this survey with a 100% response rate, with the majority of respondents being women (n=282; 75%). Most parents were aged 29-39 years (n=174; 46.27%). The highest reported level of education was college or higher (n=214; 56.91%). Seventy-three parents (19.41%) reported having a child with special needs. Only 102 parents (27.12%) reported that their children received a regular developmental assessment during vaccination visits (Table 1).

**Table 1:** Parents’ demographics ^*^ statistical significance SAR: Saudi Riyal

Variables		n (%)	Mean Knowledge	P-value
Gender				
	Men	94 (25.0)	6.04	0.0226*
	Women	282 (75.0)	6.78	
Age (years)				
	18-28	64 (17.0)	6.76	0.78
	29-39	174 (46.3)	6.65	
	40-50	123 (32.7)	6.48	
	Above 50	15 (3.99)	6.06	
Monthly Family Income				
	Less than 4000 SAR	48 (12.8)	6.16	0.52
	From 4000 to under 10000 SAR	214 (57.1)	6.67	
	From 10000 to under 20000 SAR	87 (23.2)	6.56	
	Above 20000 SAR	26 (6.93)	7.07	
Level of education				
	Lower than high school	52 (14.14)	5.15	0.0038*
	High school	108 (28.7)	6.54	
	College and higher	214 (56.9)	6.89	
Relationship status				
	Married	357 (94.95)	6.60	0.77
	Unmarried	19 (5.05)	6.42	
Number of children				
	0	33 (8.78)	6.75	0.45
	1-2	170 (45.2)	6.75	
	≥3	173 (46.0)	6.40	
Presence of child with special needs?				
	Yes	73 (19.4)	6.58	0.98
	No	303 (80.6)	6.59	
Presence of a helper at home?				
	Yes	145 (38.6)	6.58	0.94
	No	231 (61.4)	6.60	
Does your child get a developmental examination when they get their vaccinations?				
	Yes	274 (72.9)	6.66	0.75
	No	102 (27.1)	6.56	

**Figure 1:**
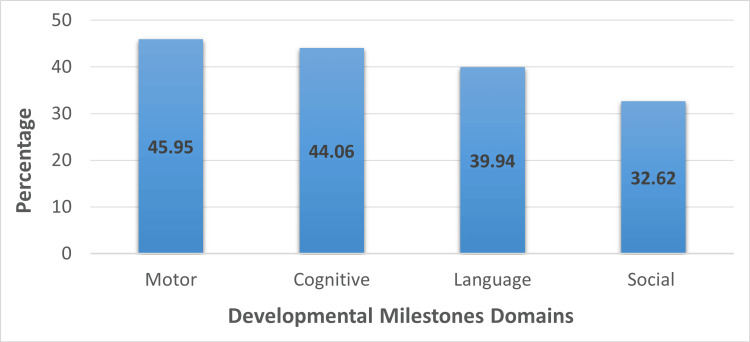
Parents’ knowledge across the different milestones domains

Out of the total number of parents surveyed, only 41 (11%) had the required level of knowledge, while the remaining 335 (89%) fell short of meeting the passing level of knowledge (mean 6.59, SD= 2.72). Parents demonstrated variable levels of knowledge across the different domains of development. The motor domain had the highest level of accuracy, followed closely behind the cognitive domain, while the language and social domains exhibited lower levels of accuracy (Figure 1).

The data presented in Table 2 and Table 3 suggest that differences in demographics did not have any statistically significant effect on the parents' knowledge level except for the parent's education level, which was the only significant factor affecting the proportion of correct answers. It is worth highlighting that the percentage of accurate responses increased with the level of education attained.

**Table 2:** Parents’ demographics and their mean knowledge scores

Parents’ Demographics		Level of Knowledge		P-value
		Failed	Passed	
Gender		n (%)	n (%)	
	Male	88 (93.62)	6 (6.38)	0.11
	Female	247 (87.59)	35 (12.41)	
Child got developmental examination when getting vaccinations				
	Yes	244 (89.05)	30 (10.95)	0.96
	No	91 (89.22)	11 (10.78)	
Children with special needs				
	Yes	65 (89.04)	8 (10.96)	0.99
	No	270 (89.11)	33 (10.89)	

**Table 3:** Parents’ demographics and their mean knowledge scores

Parents’ Demographics		Level of Knowledge		P-value
		Failed	Passed	
Level of education		n (%)	n (%)	
	College and higher degrees	183 (54.63)	31 (75.61)	0.03
	High school	100 (29.85)	8 (19.51)	
	Less than High school	52 (15.52)	2 (4.88)	
Children under the age of 14 years old				
	0 years	30 (8.96)	3 (7.32)	0.87
	1-2 years	150 (44.78)	20 (48.78)	
	3-14 years	155 (46.27)	18 (43.90)	

The majority of parents rely on online resources (54%) and their general practitioners/pediatricians (51%) for information on their children's developmental milestones. Other resources such as consulting friends and relatives and parenting books were less common sources of information among most parents, 47% and 45%, respectively (Table 4).

**Table 4:** Frequency of each source that parents rely on for information

Source	Always, n (%)	Usually, n (%)	Rarely, n (%)	Never, n (%)
General practitioner/pediatrician	190 (51.5)	112 (30.4)	29 (7.86)	38 (10.3)
Social media posts and broadcasts	74 (19.9)	92 (24.8)	108 (29.1)	97 (26.1)
Parenting seminars and courses	69 (18.6)	82 (22.2)	120 (32.4)	99 (26.8)
Relatives and friends	73 (19.7)	122 (33.0)	116 (31.4)	59 (15.9)
Internet	130 (35.4)	112 (30.5)	65 (17.7)	60 (16.3)
Television shows	68 (18.4)	114 (30.9)	113 (30.6)	74 (20.1)
Parenting books and magazines	87 (23.5)	101 (27.2)	127 (34.2)	56 (15.1)

## Discussion

Though parents' knowledge of typical trajectories of milestones development has an integral role in promoting their children's health, our study reveals that a vast majority (89%) of parents lack the necessary knowledge related to their children's development. 

In various studies conducted in both local and global contexts, it has been observed that parents have varying levels of knowledge across the different domains of milestones development. Motor development is the domain in which parents demonstrate the highest level of knowledge, followed by cognitive development. However, the social and language domains are often areas where parents display a lower level of knowledge. This trend has been observed consistently across multiple studies [9-11].

Among the potential factors that may influence the parents’ level of knowledge, the present study showed a significant association with the educational level and a positive trend towards the highest level of education. This finding contrasts with previous studies that have yielded mixed results. For instance, a study conducted to assess the mother’s developmental milestones knowledge revealed an insignificant association with their level of education [7]. Other potential culprits, such as gender and socioeconomic status, among many other variables, did not significantly impact parental knowledge in the present study. Further studies showed variable positive or negative associations. Gender, for instance, has been identified as a potential factor, with women exhibiting a more appropriate level of knowledge in one study [12].

Although having a child with special needs could potentially increase parental knowledge due to their increased exposure to medical care [8,13]. Nevertheless, no significant variation was observed within our group regarding this aspect. 

It is noteworthy that 73% of parents stated that their children in this cohort received a developmental examination during their vaccination visits, in contrast to a study conducted nationwide in the United States that found a lower rate of 30.4% [14]. Nevertheless, this was not considerably efficient to have a significant impact in this study. 

Research conducted on the Saudi Arabian population's health information-seeking habits has shown that physicians were the main source of information in some studies [10,14], while other studies suggested that the internet was the most used resource [15]. Similarly, our study found that most parents rely on either their physician or online resources for reliable health information. Hence, healthcare professionals should invest in empowering parents role in monitoring their children's growth and identify early signs of developmental delay by providing them with educational materials or reliable resources.

The study has some limitations. Firstly, the relatively small sample size may not be representative of the whole population. Also, this study is a single-center study. However, these limitations are balanced by the fact that this study is the first to be conducted in this specfic topic field. 

## Conclusions

Despite the fact that the majority of parents in this group possessed a college education and utilized multiple health resources to promote their children's development, there exists a significant gap in their knowledge. This is a cause for concern since it has become apparent in our local clinical practice that there is a presentational delay before seeking medical advice, resulting in negative consequences such as diagnostic delays and unfavorable outcomes. Additionally, most parents have reported that their children underwent a developmental screening evaluation during their vaccination visits. However, this did not influence their level of knowledge. This ought to be analyzed to assess the shortcomings in the consistency of regular practice and potentiate the parents' active role in their children's well-being. It must be emphasized that the conclusions drawn from this study are confined to a single center and are solely descriptive in character, rendering their generalizability questionable. Thus, there is an imperative need for nationwide endeavors with larger sample sizes that can aid in promoting parents' proactive role in monitoring their children's growth and encouraging them to participate in local campaigns and educational classes.
